# Variable Carbon Catabolism among *Salmonella enterica* Serovar Typhi Isolates

**DOI:** 10.1371/journal.pone.0036201

**Published:** 2012-05-25

**Authors:** Lay Ching Chai, Boon Hong Kong, Omar Ismail Elemfareji, Kwai Lin Thong

**Affiliations:** 1 Institute of Biological Sciences, Faculty of Science, University of Malaya, Kuala Lumpur, Malaysia; 2 Laboratory of Biomedical Science and Molecular Microbiology, Institute of Graduate Studies, University of Malaya, Kuala Lumpur, Malaysia; Indian Institute of Science, India

## Abstract

**Background:**

*Salmonella enterica* serovar Typhi (*S.* Typhi) is strictly a human intracellular pathogen. It causes acute systemic (typhoid fever) and chronic infections that result in long-term asymptomatic human carriage. *S.* Typhi displays diverse disease manifestations in human infection and exhibits high clonality. The principal factors underlying the unique lifestyle of *S.* Typhi in its human host during acute and chronic infections remain largely unknown and are therefore the main objective of this study.

**Methodology/Principal Findings:**

To obtain insight into the intracellular lifestyle of *S.* Typhi, a high-throughput phenotypic microarray was employed to characterise the catabolic capacity of 190 carbon sources in *S.* Typhi strains. The success of this study lies in the carefully selected library of *S.* Typhi strains, including strains from two geographically distinct areas oftyphoid endemicity, an asymptomatic human carrier, clinical stools and blood samples and sewage-contaminated rivers. An extremely low carbon catabolic capacity (27% of 190 carbon substrates) was observed among the strains. The carbon catabolic profiles appeared to suggest that *S.* Typhi strains survived well on carbon subtrates that are found abundantly in the human body but not in others. The strains could not utilise plant-associated carbon substrates. In addition, α-glycerolphosphate, glycerol, L-serine, pyruvate and lactate served as better carbon sources to monosaccharides in the *S.* Typhi strains tested.

**Conclusion:**

The carbon catabolic profiles suggest that *S.* Typhi could survive and persist well in the nutrient depleted metabolic niches in the human host but not in the environment outside of the host. These findings serve as caveats for future studies to understand how carbon catabolism relates to the pathogenesis and transmission of this pathogen.

## Introduction

The *Salmonella enterica* serovar Typhi (*S*. Typhi) is an important human intracellular pathogen of global importance, infecting as many as 21.7 million people and killing 217,000 people annually [1]. The pathogen causes typhoid fever in humans and is more prevalent in less developed countries in South Central and Southeast Asia with poor sanitation and unsafe water and food supply [1]. The key risk factors of the disease include consumption of contaminated water and food and contact with human carriers. In Malaysia, healthy human carriers were detected in Kelantan, which is a hotspot for typhoid fever in Malaysia [2]. *S*. Typhi is rarely detected in water and food. Unlike other serovars of *Salmonella*, which invade only the mucosal surface of the intestines, *S.* Typhi has evolved the ability to spread to deeper tissues, including the liver, spleen, bone marrow and gallbladder, in which it persists, multiplies and disseminates in the urine and faeces [3]. *S*. Typhi is exclusively adapted to infect human host.

Despite the unique pathology and epidemiologic characteristics of *S*. Typhi, it has received less attention globally as compared with other *Salmonella* serovars, such as *S*. Typhimurium and *S*. Enteritidis [4]. As the world continues to focus on many recent findings concerning the pathogenic and adaptive mechanisms of foodborne *S*. Typhimurium and *S*. Enteritidis, the principal factors underlying the unique epidemiological pattern and disease manifestation of this virulent, human-restricted, intracellular pathogen, *S*. Typhi, remains intruiging. In recent years, increasing evidence has implicated carbon catabolism as a virulence determinant of human pathogens [5]-[7]. The ability of pathogenic bacteria to metabolize various nutrients, especially carbon sources, is essential for the invasion, growth, survival and colonisation in intestinal and extra-intestinal sites in their hosts. To successfully colonise and persist in the various niches within the host during the course of infection, bacterial pathogens need to adjust and adapt their metabolic activity to the local nutrient availability. Nonetheless, as compared with the growing knowledge of molecular bacterial virulence and pathogenesis, research on pathogenic bacterial metabolism and persistence in the human host has progressed very little [6].

*S.* Typhi exhibits unique characteristics as an intracellular human pathogen that are not observed in other human bacterial pathogens: it is human host restricted and incapable of infecting other living organisms; able to cause both acute and chronic infection, displaying various disease manifestations; and able to transform the human host into long-term asymptomatic carriage in the environment with periodical dissemination via urine and faeces. Therefore, it is important to understand the intracellular lifestyle of this unique pathogen. To do so, we employed high-throughput phenotypic microarray analysis to characterise the carbon metabolic capacity of *S.* Typhi in the human host.

The novelty of this study is derived from the interesting and diverse background of each of the *S.* Typhi strains included in this work. These *S.* Typhi strains were carefully selected to include strains from Malaysia and Chile to determine whether there were any metabolic differences between the two areas of typhoid endemicity which are distantly separated. To explore our hypothesis that the metabolic capacity of the strains isolated from stool develops an adaptive persistence mechanism in the liver or gallbladder during chronic infection, these stool strains will differ from the strains isolated from the blood during acute systemic infection in the human host. A bacterial strain originated from a healthy human carrier was included in this study to contrast with the strains of transient chronic infection. The occurrence of *S.* Typhi in the environment is extremely rare, even though it is generally accepted that the pathogen could be transmitted via contaminated water and foods. To examine whether transient persistence in the sewage-contaminated water requires a special metabolic capacity, we have included a strain isolated from sewage-contaminated river in Chile during the 1983 Chilean Typhoidal outbreak [8].

## Results and Discussion

### Phenotypic Characteristics

The eight unique *S.* Typhi strains studied in this work were characterised into six pulsotypes based on pulsed-field gel electrophoresis, which was performed previously in the laboratory (unpublished data). Of these strains, ST191/05 and ST196/05, both from the Kelantan outbreak in 2005, shared a similar pulsotype. Similarly, strains ST5680/07, from another outbreak in 2007, and STC63/07 were isolated from a healthy human carrier in the same locality, were and found to display identical pulsotypes. This observation led to the speculation that the chronic human carriage acts as the pathogen reservoir in this hyperendemic state. However, the virulotyping of 22 virulence and virulence-associated genes clearly distinguished the outbreak strain ST5680/07 from the carrier strain, STC63/07, which was also grouped together with ST33/06 (Table 1). Further analysis of motility and carbon catabolism revealed divergent responses for these two strains, therefore suggesting that ST5680/07 and STC63/07 are two unique strains. However, strains ST191/05 and ST196/05 with similar pulsotype also shared similar virulotypes, motility characteristic and carbon catabolic profiles. Undoubtedly, ST191/05 and ST196/05 possibly originated from the same ancestral strain.

**Table 1:** *Salmonella enterica* serovar Typhi strains used in this study.

Strain	Year	Source	Locality	Pulsotype	Virulotype*	Motility
ST191/05	2005	Human (blood); outbreak	Kelantan, Malaysia	STXba003	V1	+++
ST196/05	2005	Human (blood); outbreak	Kelantan, Malaysia	STXba003	V1	+++
ST5680/07	2007	Human (stool); outbreak	Kelantan, Malaysia	STXba004	V3	+++
STC63/07	2007	Healthy human carrier (stool)	Malaysia	STXba004	V2	+
ST33/06	2006	Human (blood); outbreak	Kelantan, Malaysia	STXba005	V2	+++
ST280/90	1990	Human (stool);outbreak	Johor, Malaysia	STXba006	V4	++
STVC1681	1983	Sewage contaminated water	Chile	STXba001	V5	+++
STVC3121	1983	Human (blood); outbreak	Chile	STXba002	V1	+++

* Virulence genes profiles among 8 *S.* Typhi strains tested.

(V) : *agf*A-*agf*C-*cdt*B-*inv*A-*iro*N-*lpf*A-*lpf*C-*mgt*C-*mis*L-*orf*L-*pef*A-*pip*D-*prg*H-*sef*C-*sef*D-*sif*A-*sit*C-*sop*B-*sop*E-*spi*C-*spv*B-*spv*C.

(V1): *agf*A-*agf*C-*cdt*B-*inv*A-*iro*N- -*mgt*C-*mis*L-*orf*L- -*pip*D-*prg*H-*sef*C-*sef*D-*sif*A-*sit*C-*sop*B-*sop*E-*spi*C-.

(V2): *agf*A-*agf*C-*cdt*B-*inv*A-*iro*N- -*mgt*C-*mis*L-*orf*L- -*pip*D-*prg*H-*sef*C- -*sif*A-*sit*C-*sop*B-*sop*E-*spi*C-.

(V3): *agf*A-*agf*C-*cdt*B-*inv*A-*iro*N- -*mgt*C-*mis*L-*orf*L- -*pip*D-*prg*H- -*sif*A-*sit*C-*sop*B-*sop*E-*spi*C-.

(V4): *agf*A-*agf*C-*cdt*B-*inv*A-*iro*N- -*mis*L-*orf*L- -*pip*D-*prg*H- -*sif*A-*sit*C-*sop*B-*sop*E-*spi*C-.

(V5): *agf*A- -*cdt*B-*inv*A-*iro*N- -*mgt*C-*mis*L-*orf*L- -*pip*D-*prg*H-*sef*C- -*sif*A-*sit*C-*sop*B-*sop*E-*spi*C-.

Fimbrial genes are located in the chromosome and on the plasmid. The *agf*A, *agf*C, *lpf*A, *lpf*C, *sef*C, and *sef*D and *pef*A genes are important for adherence to different sites of the host cells; any loss of these fimbrial genes will decrease the ability of *Salmonella* for adherence in the host cells [9], [10]. The *S*. Typhi strains tested lacked plasmids (data not shown); thus, it was not surprising that the *spvB* and *spv*C, *pef*A, *lpf*A and *lpf*C genes, which are located on the virulence plasmid, were absent. Nevertheless, variations among the strains were observed in the aggregative fimbriae gene (*agf*C), the SEF14 fimbriae genes *(sef*C and *sef*D) and the genes involved in the intra-macrophage survivability (*mgt*C). The strains ST190/07 and ST196/07, derived from the 2007 typhoid outbreak, and strain STVC3121, derived from Chilean typhoid outbreak, possessed 17 out of 22 virulence and virulence-associated genes tested. The fimbriae genes, *sef*C and *sef*D, were not detected in strains ST5680/07 and ST280/90. In addition, *mgt*C gene was absent in strain ST280/90. The aggregative fimbriae gene (*agf*C) was detected in all strains except the Chilean environmental strain, STVC1681. The virulotype profiles are shown in Table 1.

We have observed remarkably little motility in the carrier strain, STC63/07, while strain ST5680/07 demonstrated rapid swarming on the agar surface (Table 1). The low motility rate was also recorded in the Johor strain, ST280/90, which was isolated from stool (Table 1). In fact, motility had been identified as a bacterial virulence factor that aids in the gut colonisation to initiate infection in the human host. The observation of a relatively weaker motility capacity in the chronic carrier strain suggests that motility might not be essential for the long-term persistence in the human host. This postulation requires further investigation to better understand the adaptive life style of *S.* Typhi within the human host.

### Limited Carbon Catabolism Capacity among *S.* Typhi Strains

**Figure 1:**
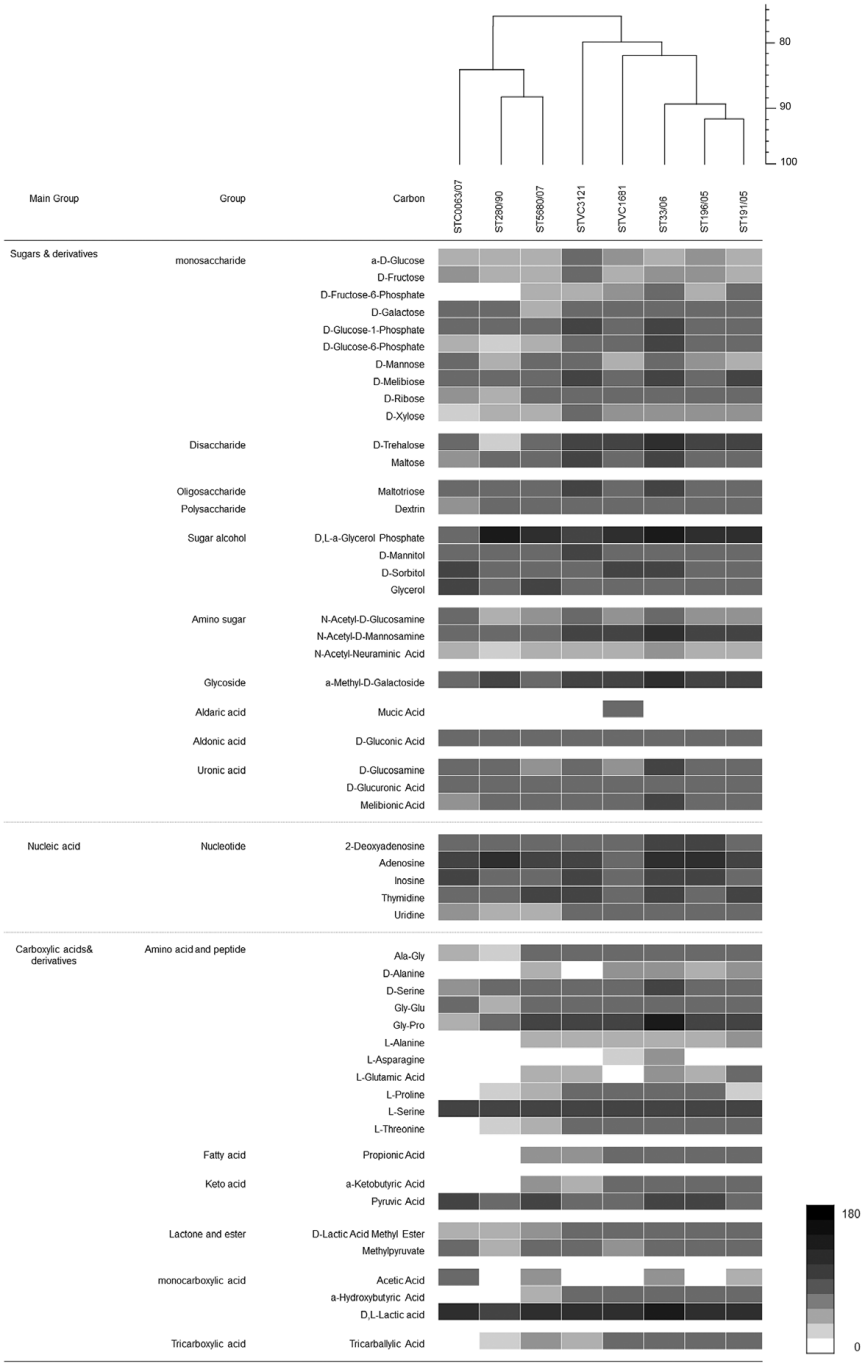
Cluster analysis based on the carbon catabolism profile of the eight *Salmonella enterica* serovar Typhi strains. The dendrogram and catabolic profiles show 52 active carbon substrates catabolised by the eight strains of *S.* Typhi tested. The cluster analysis was performed with simple matching similarity based on the catabolic profile of 190 carbon substrates and the dendrogram was built with unweighted paired group of arithmetic mean (UPGMA).

**Figure 2:**
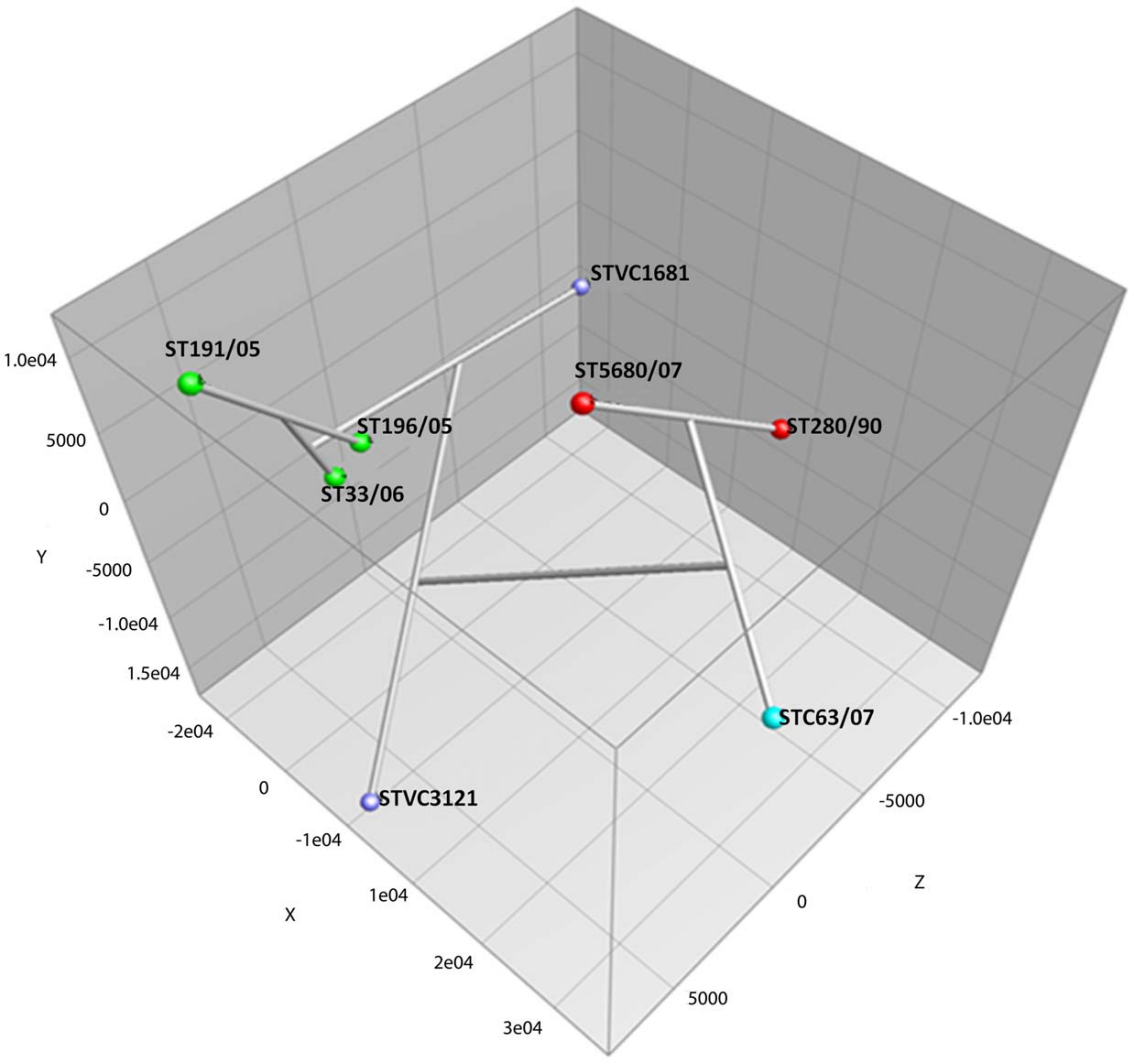
Three-dimensional principle component analysis (PCA) of eight *Salmonella enterica* serovar Typhi strains. A three-dimensional principle component analysis (PCA) of the eight *S.* Typhi strains was calculated from the corrected average area under the bacterial growth curve. For each carbon well, the average area under the bacterial growth curve was corrected to zero if the T_max_–T_min_ value was lesser than the threshold value of 50.

The carbon catabolism capacity of *S.* Typhi strains tested was extremely low, recording only 21% to 27% of 190 carbon substrates tested (Figure 1, Table S1). As compared with other environmental bacteria and pathogens, such as *S.* Typhimurium [11], *Pseudomonas aeruginosa*, and *Burkholderia cepacia*, that are able to grow on more than 60 different types of carbon substrates, *S.* Typhi was metabolically less versatile. Of these 27% (52 carbon substrates) of utilisable carbon substrates, half (27 out of 52 substrates) were carbohydrates; 20% were carboxylic acids, including amino acid, peptide and fatty acid; and 10% were nucleosides (Table 2, Table S2). Almost all of the carbon substrates utilisation by the strains is exclusively available in the gut, liver, bile and blood of human host. Plant-derived carbon sources, such as arbutin, gelatin, pectin, mannan, palatinose, xylitol and sorbic acid, were not able to support growth in the *S.* Typhi strains tested. The limited catabolic activity of the only carbon sources available in the human host might be an evidence for the restricted infection of *S.* Typhi to only human host and that the pathogen can hardly persist in the environment outside of the host. While we expected to observe a somewhat higher carbon catabolism in the only environmental strain included in this study (STVC1681), the PM profiling did not reveal a significantly wider range of carbon source utilisation. Nonetheless, the carbon catabolic profile of this environmental strain was unique, as shown in the PCA analysis (Figure 2). The strain STVC1681 was isolated from the summer typhoid outbreak in 1983 from the sewage contaminated Mapocho River, Santiago, Chile [8], and was the only strain that could respire on mucic acid. According to the classical work of Sternfeld and Saunders [12], *S.* Enteritidis, and *S.* Schottmiilleri utilised mucic acid for the production of acid; *Aerobacter aerogenes* and *S. aertrycke* produced acid and gas; and *Eberthella typhi* (was renamed as *S.* Typhi), *Shigella dysenteriae*, *S.* Choleraesuis, and *S.* Paratyphi did not respire on mucic acid at all [13]. It is interesting to further investigate whether the ability to utilise mucic acid contributes to the survival *S.* Typhi outside of human host. According to the classical study on carbon catabolism of *S.* Typhimurium conducted by Gutnick and co-workers [11] in 1969, *S.* Typhimurium, a less-specialised pathogen, could utilise 73 carbon sources tested. In comparison, almost all carbon sources utilised by *S.* Typhi in this study were reported to be catabolised by *S.* Typhimurium LT-2, including mucic acid. However, not all compounds used by *S.* Typhimurium (about 15 compounds) [11] could serve as the sole carbon sources for *S.* Typhi. Perhaps, the capability to catabolise a wider range of carbon sources is an essential property for bacterial survival in a broader host range or environment. Nonetheless, this hypothesis requires further investigation.

**Table 2:** Catabolism of 190 carbon substrates by *Salmonella enterica* serovar Typhi strains.

Carbon substrate		*No. of carbonsubstrates catabolisedby all strains	*No. of carbon substrates catabolised by at least a strain	*No. of carbon substrates not catabolised	*No. of carbon substrates tested
Sugars and derivatives	Monosaccharide	9(45)	1(5)	10(50)	20
	Disaccharide	2(22)		7(78)	9
	Oligosaccharide	1(10)		9(90)	10
	Polysaccharide	1(17)		5(83)	6
	Sugar alcohol	4(22)		14(78)	18
	Amino sugar	3(38)		5(63)	8
	Deoxy sugar			4(100)	4
	Aldaric acid		1(20)	4(80)	5
	Aldonic acid	1(50)		1(50)	2
	Uronic acid	3(38)		5(63)	8
	Glycoside	1(11)		8(89)	9
Carboxylic acids and derivatives	Monocarboxylic acid	1(11)	2(22)	6(67)	9
	Dicarboxylic acid			11(100)	11
	Tricarboxylic acid		1(33)	2(67)	3
	Keto acid	1(11)	1(11)	7(78)	9
	Fatty acid		1(14)	6(86)	7
	Amino acid and peptide	5(15)	6(18)	22(67)	33
	Amide			3(100)	3
	Ketone			3(100)	3
	Lactone and ester	2(40)		3(60)	5
	Polysorbate surfactant			3(100)	3
Nucleic acid	Nucleotide	5(100)			5
	**TOTAL**	**39(21)**	**13(7)**	**138(73)**	**190**

* Number of substrate catabolised by all strains or some strains or not able to be catabolised by any strains (percentage of substrate catabolised by all strains or some strains or not able to be catabolised by any strains).

*S.* Typhi strain ST280/90 demonstrated extremely low carbon metabolic activity. Virulotyping revealed ST280/90 as a variant strain lacking *mgtC* gene. *mgt*C encodes for a 25-kDa protein of unknown function. In *S*. Typhimurium [14] and *S*. Typhi [15], the experimental evidence suggested that *mgt*C participates in the adaptation to low-Mg^2+^ environments, supporting bacterial invasion and proliferation in macrophages. MgtC is a virulence factor that plays a vital role that is not supplied by any other bacterial factor located in *Salmonella* Pathogenicity Island-3 (SPI-3) [15]. The related studies have shown that the *Salmonella* mutant lacking *mgtC* grew significantly less than the wild type at 10 µM of MgCl_2_ whereas no difference was observed between the mutant and wild type at 10 mM Mg^2+^ [15]. Therefore, we suspected that the concentration of MgCl_2_ (50 µM) in the Biolog inoculating fluid, IF-0 [16], might be too low to promote maximum growth of the variant strain. To clarify the speculation, we cultivated the variant strain, ST280/90, in M9 minimal media supplemented with glucose and 10 µM, 50 µM and 100 µM of MgCl_2_. We observed no growth in all the three MgCl_2_ concentrations tested (data not shown). Despite its poor growth in most carbon sources, we find it rather intriguing that ST280/90 showed comparable growth as with the other seven strains in glycerol, a-methyl-D-galactoside, 2-deoxyadenosine, adenosine and L-serine (Figure 1). Further investigation is needed to find out how these carbon sources compensate for the lack of *mgt*C gene, particularly the *S*. Typhi strain.

### *S.* Typhi Showed Active Respiration on Nucleosides, α-glycerolphosphate, Glycerol, L-serine, Pyruvate and Lactate

*S.* Typhi strains were able to uptake and metabolise most of the monosaccharides tested, such as D-glucose, D-fructose, D-galactose and D-glucose-1-phosphate. However, relative to the respiration rate on nucleosides, α-glycerolphosphate, glycerol, L-serine, pyruvate and lactate, monosaccharides supported only moderate respiration in *S.* Typhi strains (Figure 1). It was not surprising that nucleosides were rapidly catabolised by *S.* Typhi strains, as numerous studies have reported that nucleosides are excellent carbon sources in *E. coli* and *Salmonella* [17],[18]. We believe that the active metabolism of nucleosides supports the rapid proliferation of *S.* Typhi during invasive infection of the bloodstream. Human blood serum is abundant in various metabolites, including various amino acids and nucelosides [19], [20]. The efficient uptake and catabolism of nucleosides during the infection process provides sufficient substrates for biosynthesis of nucleotide bases required for DNA synthesis and repair of damaged DNA induced by the host’s reactive oxygen intermediates during infection [21].

Alpha-glycerolphosphate and glycerol are abundant in the liver and kidneys. In the human body, glycerol is rapidly adsorbed in the intestines and stomach and distributed over the extracellular space [22]. Glycerol is phosphorylated to α-glycerophosphate by glycerol kinase, predominantly in the liver (80–90%) and kidneys (10–20%) [22]. Glycerol, L-serine, pyruvate and lactate serve as the substrate or end product of gluconeogenesis and glycolysis, which also occurs predominantly in the liver and to a lesser extend in the cortex of kidney. Therefore, the observation of the relatively active metabolism of these carbon sources was highly speculated to be associated to adaptive mechanisms acquired by *S.* Typhi for the colonisation and long-term persistence in the human host, specifically in the liver, during chronic infection in the human host. The availability of α-glycerophosphate, glycerol, L-serine, pyruvate and lactate in the cortex of the kidney suggested the possible transient colonisation of *S.* Typhi in human kidneys and causes the dissemination of *S.* Typhi via urine. In fact, it has been known for years that the typhoid human carriers disseminate the pathogen through faeces and urine. This observation will be investigated more in depth in a future study.

### Distinct Carbon Catabolism Profile between Strains Isolated from Human Stools and Blood

PCA analysis exhibited clearly distinct carbon catabolic activity between the two clusters of *S.* Typhi strains: ST191/05, ST196/05 and ST33/06 as compared with ST5680/07, ST280/90 and STC63/07 (P>0.05) (Table 3). The differences observed appeared to be associated with the origin of the strains. The strains ST191/05, ST196/05 and ST33/06 were all isolated from human blood samples, sharing a similarity of 86.7% to 91.2% in their carbon catabolic profile whereas the catabolic activity among the strains obtained from stool samples (ST280/90, ST5680/07 and STC63/07) was more diverse, with a lower similarity level, which ranged from 82.5% to 87.6%. Although the clinical Chilean strain STVC3121 was also isolated from human blood, it was catabolically unique from both clusters and from the environmental Chilean strain (STVC1681). Notably, all the three strains derived from the stool samples had a narrower range in carbon utilisation and were comparatively weaker in their catabolic activity (P<0.05; Table 3), specifically in the catabolism of uridine and D-glucose-6-phosphate. The motility test and growth in rich medium confirmed the findings discussed above, as both analyses demonstrated lower motility and delayed growth in *S*. Typhi strains from stools. The ability to catabolise glucose-6-phosphate has been associated with the intracellular survival and virulence of *Salmonellae* in mice [23]. This observation was indeed intriguing, as *S.* Typhi strains isolated from stool and blood actually represented two different stages in human infection and colonisation niches within the human body. *S.* Typhi is able to invade the intestinal wall and replicate within macrophages and infected phagocytes [24]. The replication of the bacteria within macrophages in the liver and spleen [25], [26] resulted in the release of the pathogen into the bloodstream [27]. The pathogen later invades the gallbladder and leads to bacterial shedding in the urine and faeces in the chronic carriage of infected individuals [28], [29]. It is possible that *S*. Typhi acquires different metabolic activity and phenotypes for colonisation and persistence in these two different niches, liver and spleen, which disseminate *S*. Typhi into the blood stream and the gallbladder, which releases *S*. Typhi in the urine and faeces. However, we could not entirely rule out the possibility that the strains could be grouped simply by the predominant strain in a given time and place. That is, the strains from 2005 and 2006 from Kelantan could simply be fortuitously similar whereas those strains isolated from 2007 would be similar; these strains would also be similar to those isolated in 1990 and the Chilean strains would comprise their own group. This grouping could be addressed through the study of more strains, preferably from the same outbreak, isolated both from blood and stools. A comprehensive study would address the phenotypic differences in *S*. Typhi.

**Table 3:** Carbon catabolic activity among *Salmonella enterica* serovar Typhi strains isolated from human blood, stool and sewage-contaminated water.

Origin	Strain	No. of carbonsubstrates utilised	Catabolism rate			Catabolism activity*
			Range	Average	Standard deviation	
Blood†	ST191/05	51	24–173	96	35	4919
	ST196/05	50	26–160	90	33	4503
	ST33/06	52	31–141	79	27	4097
	STVC3121	49	24–248	90	32	4418
Stool†	STC63/07	40	12–139	74	32	2940
	ST280/90	43	3–138	56	33	2392
	ST5680/07	51	28–155	74	33	3753
Sewage contaminated water	STVC1681	51	15–151	88	31	4482
**TOTAL**		**48**	**3–248**	**81**	**32**	**3938**

* Catabolism activity = No. of carbon sources utilized × mean of catabolism rate.

† Catabolism activity between strains of blood origin and stool origin were significantly different at P-value of 0.013 (exclude the environmental strain VC1681) and 0.029 (include strain VC1681).

### High Divergence in Carboxylic Acids Catabolism

Another notable finding in this study was the observation of great divergence in the catabolism of amino acids, fatty acids and other carboxylic acids. While less variation was recorded in the metabolism of sugars, one strain of *S.* Typhi was unable to grow on these six amino acids (L-proline, D-alanine, L-alanine, L-glutamic acid, L-asparagine and L-threonine) and three short-chain fatty acids (propionic acid, a-ketobutyric acid and a-hydroxybutyric acid). The phenotypic microarray analyses revealed that both the carrier (STC63/07) and the Johor strain (ST280/90) lacked the catabolic pathways to uptake and assimilate most of the amino acids and fatty acids stated above. Studies have shown that the ability to catabolise amino acids in *Salmonella* is essential for the virulence in mice [30]–[33]. In addition, the catabolism of C2 substrates (e.g., fatty acids) plays a crucial role in *Salmonella* metabolism *in-vivo* as the mutants of *S*. Typhimurium that had lost their ability to grow on C2 substrates are attenuated in mice [34]. The ability to metabolise on fatty acids might also play a role in the early stages of infection, when the bacteria reside within the lipid-rich mucus covering the intestinal epithelium [35]. Metabolism of short chain fatty acids might also be involved in the signaling required for expression of virulence genes or the induced detoxification of these compounds, which can have antimicrobial effects at high concentrations [35]–[37]. Additional work is needed to determine how the ability to catabolise carbon substrates relates to the virulence of *S*. Typhi *in-vivo*.

### Conclusion and Future Directions

In conclusion, the findings obtained in this study have revealed interesting variations in the carbon catabolism among the *S.* Typhi strains of a different background. The observation was indeed astonishing, as carbon catabolism is one of the earliest means used by the scientists to distinguish the bacteria, and is an essential property of bacterial survival and persistence in its specific niches of habitat. The diversity observed in the carbon catabolism profiles among this set of diverse *S*. Typhi strains has suggested the possible involvement of various metabolic pathways that might be related to the virulence and pathogenesis of this host-restricted human pathogen. The findings obtained in this study would serve as an important foundation for future genomic and transcriptomic studies to understand the relationship of carbon metabolism to the pathogenesis and transmission of this human-host-adapted pathogen. The questions concerning the diversity of carbon catabolism provide caveats for the future study of the pathogenicity of *S*. Typhi.

## Materials and Methods

### Media and Strains Used

Eight clinical strains of *S.* Typhi from our culture library were selected for the present work (Table 1). All strains, except STVC1681 and STVC3121 from the 1983 typhoid outbreak in Chile [38] were of local origin. Four *S*. Typhi strains, namely ST191/05, ST196/05, ST33/06 and ST5680/07, were isolated during the typhoid outbreaks in the northeast state of Peninsular Malaysia, Kelantan, which was a major typhoid hotspot from 2005 to 2007. The former two strains were isolated from blood samples in 2005 and were genetically identical in terms of PFGE subtyping; ST33/06 was isolated from a blood sample in 2006, and ST5680/07 was isolated from a stool sample in 2007. In 2007, a survey was performed in Kelantan to detect the presence of potential typhoid carrier. Strain STC63/07 was isolated from the stool sample of a healthy adult in Kelantan. The pulsotype of STC63/07 was indistinguishable from strain ST5680/07, which was isolated from the 2007 Kelantan typhoid outbreak (unpublished data). *S*. Typhi strain ST280/90 originated from the stool sample of a victim of another typhoid outbreak in Malaysia, Johor, in 1990 and was also included in the study [38]. All *S.* Typhi strains were cultured and maintained on Luria-Bertani (LB) medium.

### Motility Test

The motility tests were performed as previously described [6]. Briefly, bacteria were grown on LB agar, transferred with an inoculation needle to the centre of an LB agar plate containing 0.3% agar and incubated for 24 hours at 37°C. The plates were measured for the colony diameter at 6, 12, 18 and 24 hours. The motility test was performed in triplicate.

### Virulotyping

Five multiplex PCRs were used to amplify the 22 genes: *agf*A, *sef*C and *sef*D [10]; *cdt*B, *iro*N, *lpf*C, *pef*A, *prg*H, *sif*A, *sit*C, *sop*B and *spv*B [39]; *mis*L, *orf*L, *pip*D, *sop*E and *spi*C [40]; *inv*A [41]; *mgt*C [42]; *agf*C [43]; *spv*C [44]; and *lpf*A [10]. These genes are involved in adhesion, invasion, intracellular survival, systemic infection, toxin production and Mg^2+^ and iron uptake. The amplification was performed in a 25 µl reaction mixture that included 100 ng of DNA template, 3 mM MgCl_2_, 0.75 U of *Taq* DNA Polymerase (Promega Inc., Madison, WI, U.S.A.), 1X PCR buffer, 400 µM dNTPs mix and 0.4 µM of each primer. The amplification was carried out in a Mastercycler Gradient (Eppendorf, U.S.A.) with the following cycling conditions: 95°C for 5 min, followed by 30 cycles of 94°C for 30 sec, 56.3°C for 30 sec, 72°C for 2 min, and a final cycle of 72°C for 10 min.

### Phenotypic Microarray

The eight strains of *S.* Typhi were assayed for the carbon utilisation on Phenotype Microarray (PM) (Biolog) microplates PM1 and PM2, and the catabolism capacity of 190 different carbon substrates was tested. The PM technology uses the irreversible reduction of tetrazolium violet to formazan as a reporter of active metabolism [16]. The reduction of the dye causes the formation of a purple colour that is recorded by a charge coupled-device camera every 15 min for the duration of the incubation period. All procedures were performed as indicated by the manufacturer. First, *S.* Typhi isolates were streaked on LB agar and incubated at 37°C for 18 hours. Five to ten single colonies of each of the isolates were carefully picked up with a moistened cotton swab and resuspended in 15 ml of Biolog inoculating fluid IF-0 until a cell density of 85% transmittance was reached. Subsequently, 1% Biolog dye A (vol/vol) was added to the cell suspension, and 100 µl of the mixture was loaded into each well on PM microplate PM1 and PM2. All PM microplates were incubated at 37°C in an OmniLog reader, and the colour changes in the wells were monitored automatically every 15 min. The readings were recorded for 24 hours, and the data were analysed using OminoLog PM software, which generated a time course kinetic curve for tetrazolium colour development. The option of A1 zero was selected during data processing to deduct the background signal with reference to the A1 negative control well in each plate. Each strain was analysed in duplicate, and the results were checked for consistency.

### Statistical Analysis of PM Results

For each well, the averaged product of the average area under the kinetic growth curve and the difference (T_max_–T_min_) of the kinetic data obtained in duplicate experiments were used to assay for positive growth under various PM conditions. The values of average area under the kinetic growth curve for all negative wells were analysed to obtain the background value so that a threshold can be determined to offset the average area obtained for each of the carbon substrates. A threshold value of 50 was set. The PM conditions exceeding a threshold value of 50 were further screened at the T_max_–T_min_ value. Double screening for both the average area and the differences ensured that only wells with an increasing signal and a sufficient total area represent a positive growth condition. The numerical values of the average area for positive PM conditions were used for further analysis with BioNumerics software (Applied Math, Kortrijk, Belgium).

For principal component analysis (PCA), the PM data were filtered using the corrected average growth area as a parameter and subsequently processed with BioNumerics software. For each well, the average growth was corrected to zero if the difference was less than the threshold value of 50. Clustering analysis was performed to group the strains based on the metabolic diversity with the simple matching similarity, and the clustering was based on UPGMA.

## Supporting Information

Table S1 Standardized value of the average area under the kinetic growth curve (catabolic rate) for each of the fifty-two carbon substrates catabolised by the *S*. Typhi strains.
(PDF)

Table S2 One-hundred and ninety various carbon sources included in the carbon metabolic profiling of the eight *S*. Typhi strains. Fifty-two substrates were utilized by the *S*. Typhi strains tested while the others were not supportive of growth of *S*. Typhi.
(PDF)
