# Supplementary material for: Variable Carbon Catabolism among Salmonella enterica Serovar Typhi Isolates
Source: PLoS One. 2012 May 25;7(5):e36201. doi: 10.1371/journal.pone.0036201 (PMC3360705; doi:10.1371/journal.pone.0036201)
Supplement: Table S1 — Standardized value of the average area under the kinetic growth curve (catabolic rate) for each of the fifty-two carbon substrates catabolised by the S. Typhi strains. (PDF) [file pone.0036201.s001.pdf]

**SUPPLEMENTARY DATA**

Standardised value of averaged area under the growth curve of 52 carbon substrates utilised by tested Salmonella Typhi strains.

| Main Group | Group | Carbon | CR0063/07 | ST280/90 | S5680/07 | VC3121 | VC1681 | 33/06 | BL 196/05 | BL 191/05 |
|---|---|---|---|---|---|---|---|---|---|---|
| Sugars & derivatives | monosaccharide | a-D-Glucose | 37 | 35 | 32 | 69 | 40 | 34 | 40 | 36 |
| | | D-Fructose | 46 | 20 | 33 | 75 | 35 | 46 | 40 | 37 |
| | | D-Fructose-6-Phosphate | 0 | 0 | 24 | 33 | 47 | 86 | 34 | 50 |
| | | D-Galactose | 52 | 52 | 33 | 78 | 64 | 66 | 59 | 51 |
| | | D-Glucose-1-Phosphate | 74 | 55 | 72 | 104 | 98 | 101 | 93 | 83 |
| | | D-Glucose-6-Phosphate | 26 | 14 | 32 | 68 | 86 | 100 | 68 | 67 |
| | | D-Mannose | 53 | 22 | 50 | 66 | 35 | 73 | 42 | 37 |
| | | D-Melibiose | 88 | 78 | 81 | 109 | 87 | 116 | 90 | 100 |
| | | D-Ribose | 42 | 34 | 51 | 83 | 61 | 72 | 66 | 66 |
| | | D-Xylose | 11 | 27 | 37 | 60 | 40 | 44 | 43 | 44 |
| | Disaccharide | D-Trehalose | 78 | 3 | 76 | 119 | 106 | 136 | 113 | 109 |
| | | Maltose | 46 | 65 | 69 | 103 | 88 | 100 | 85 | 84 |
| | Oligosaccharide | Maltotriose | 86 | 69 | 71 | 102 | 72 | 111 | 77 | 76 |
| | Polysaccharide | Dextrin | 45 | 82 | 70 | 80 | 80 | 95 | 85 | 73 |
| | Sugar alcohol | D,L-a-Glycerol Phosphate | 98 | 144 | 127 | 109 | 121 | 142 | 137 | 124 |
| | | D-Mannitol | 75 | 66 | 60 | 102 | 81 | 93 | 74 | 68 |
| | | D-Sorbitol | 103 | 85 | 88 | 90 | 109 | 103 | 99 | 84 |
| | | Glycerol | 109 | 92 | 110 | 67 | 69 | 88 | 77 | 80 |
| | Amino sugar | N-Acetyl-D-Glucosamine | 64 | 36 | 45 | 81 | 45 | 50 | 49 | 41 |
| | | N-Acetyl-D-Mannosamine | 85 | 86 | 77 | 115 | 104 | 127 | 118 | 107 |
| | | N-Acetyl-Neuraminic Acid | 32 | 10 | 32 | 36 | 22 | 43 | 30 | 24 |
| | Glycoside | a-Methyl-D-Galactoside | 82 | 102 | 91 | 109 | 110 | 125 | 114 | 111 |
| | Aldaric acid | Mucic Acid | 0 | 0 | 0 | 0 | 90 | 0 | 0 | 0 |
| | Aldonic acid | D-Gluconic Acid | 67 | 71 | 73 | 96 | 76 | 84 | 80 | 78 |
| | Uronic acid | D-Glucosamine | 70 | 54 | 49 | 95 | 47 | 109 | 64 | 63 |
| | | D-Glucuronic Acid | 88 | 69 | 94 | 92 | 83 | 81 | 87 | 79 |
| | | Melibionic Acid | 41 | 68 | 78 | 71 | 88 | 106 | 85 | 80 |
| Nucleic acid | Nucleotide | 2-Deoxyadenosine | 94 | 88 | 78 | 60 | 74 | 101 | 105 | 96 |
| | | Adenosine | 114 | 126 | 114 | 106 | 85 | 123 | 125 | 110 |
| | | Inosine | 107 | 83 | 96 | 109 | 96 | 108 | 102 | 93 |
| | | Thymidine | 87 | 82 | 100 | 100 | 90 | 111 | 97 | 106 |
| | | Uridine | 48 | 36 | 36 | 80 | 64 | 79 | 75 | 64 |
| Carboxylic acids& derivatives | Amino acid and peptide | Ala-Gly | 25 | 14 | 55 | 56 | 54 | 69 | 55 | 65 |
| | | D-Alanine | 0 | 0 | 32 | 0 | 42 | 41 | 39 | 46 |
| | | D-Serine | 47 | 69 | 61 | 92 | 98 | 104 | 99 | 82 |
| | | Gly-Glu | 74 | 32 | 55 | 53 | 76 | 92 | 80 | 69 |
| | | Gly-Pro | 29 | 80 | 100 | 106 | 113 | 141 | 114 | 105 |
| | | L-Alanine | 0 | 0 | 28 | 21 | 36 | 38 | 30 | 47 |
| | | L-Asparagine | 0 | 0 | 0 | 0 | 13 | 44 | 0 | 0 |
| | | L-Glutamic Acid | 0 | 0 | 25 | 23 | 0 | 41 | 22 | 60 |
| | | L-Proline | 0 | 13 | 33 | 80 | 81 | 92 | 75 | 18 |
| | | L-Serine | 100 | 100 | 100 | 100 | 100 | 100 | 100 | 100 |
| | | L-Threonine | 0 | 12 | 32 | 51 | 80 | 95 | 67 | 75 |
| | Fatty acid | Propionic Acid | 0 | 0 | 47 | 47 | 57 | 81 | 70 | 80 |
| | Keto acid | a-Ketobutyric Acid | 0 | 0 | 48 | 36 | 74 | 57 | 56 | 66 |
| | | Pyruvic Acid | 109 | 67 | 102 | 96 | 96 | 116 | 105 | 98 |
| | Lactone and ester | D-Lactic Acid Methyl Ester | 33 | 28 | 42 | 56 | 67 | 86 | 75 | 58 |
| | | Methylpyruvate | 50 | 32 | 66 | 69 | 46 | 58 | 57 | 57 |
| | monocarboxylic acid | Acetic Acid | 55 | 0 | 46 | 0 | 0 | 44 | 0 | 27 |
| | | a-Hydroxybutyric Acid | 0 | 0 | 35 | 57 | 74 | 76 | 72 | 82 |
| | | D,L-Lactic acid | 127 | 115 | 134 | 128 | 127 | 157 | 135 | 131 |
| | Tricarboxylic acid | Tricarballylic Acid | 0 | 16 | 47 | 28 | 66 | 82 | 71 | 68 |
