# Supplementary material for: Variable Carbon Catabolism among Salmonella enterica Serovar Typhi Isolates
Source: PLoS One. 2012 May 25;7(5):e36201. doi: 10.1371/journal.pone.0036201 (PMC3360705; doi:10.1371/journal.pone.0036201)
Supplement: Table S2 — One-hundred and ninety various carbon sources included in the carbon metabolic profiling of the eight S. Typhi strains. Fifty-two substrates were utilized by the S. Typhi strains tested while the others were not supportive of growth of S. Typhi. (PDF) [file pone.0036201.s002.pdf]

Carbon substrates catabolised by *S.* Typhi strains

2-Deoxyadenosine	D-Glucose-6-Phosphate	L-Proline
Acetic Acid	D-Glucuronic Acid	L-Serine
Adenosine	D-Lactic Acid Methyl Ester	L-Threonine
a-D-Glucose	D-Mannitol	Maltose
a-Hydroxybutyric Acid	D-Mannose	Maltotriose
a-Ketobutyric Acid	D-Melibiose	Melibionic Acid
Ala-Gly	D-Ribose	Methylpyruvate
a-Methyl-D-Galactoside	D-Serine	Mucic Acid
D,L-a-Glycerol Phosphate	D-Sorbitol	N-Acetyl-D-Glucosamine
D,L-Lactic acid	D-Trehalose	N-Acetyl-D-Mannosamine
D-Alanine	D-Xylose	N-Acetyl-Neuraminic Acid
Dextrin	Glycerol	Propionic Acid
D-Fructose	Gly-Glu	Pyruvic Acid
D-Fructose-6-Phosphate	Gly-Pro	Thymidine
D-Galactose	Inosine	Tricarballylic Acid
D-Gluconic Acid	L-Alanine	Uridine
D-Glucosamine	L-Asparagine	
D-Glucose-1-Phosphate	L-Glutamic Acid	

Carbon substrates cannot be catabolised by *S.* Typhi strains

1,2-Propanediol	D-Fucose	L-Isoleucine
2,3-Butanediol	D-Galactonic Acid-g-Lactone	L-Leucine
2,3-Butanone	D-Galacturonic Acid	L-Lysine
2-Aminoethanol	D-Glucosaminic Acid	L-Lyxose
2-Deoxy-D-Ribose	Dihydroxyacetone	L-Malic Acid
2-Hydroxybenzoic acid	D-Lactitol	L-Methionine
2-Oxovaleric acid	D-Malic Acid	L-Ornithine
3-0-beta-D-Galactopyranosyl-D-Arabinose	D-Melezitose	L-Phenylalanine
3-Hydroxy 2-Butanone	D-Psicose	L-Pyroglutamic Acid
3-Methylglucose	D-Raffinose	L-Rhamnose
4-Hydroxybenzoic Acid	D-Ribono-1,4-Lactone	L-Sorbose
5-Keto-D-Gluconic Acid	D-Saccharic Acid	L-Tartaric Acid
Acetamide	D-Tagatose	L-Valine
Acetoacetic Acid	D-Tartaric Acid	Malonic Acid
a-Cyclodextrin	D-Threonine	Maltitol
a-D-Lactose	Dulcitol	Mannan
Adonitol	Formic Acid	m-Hydroxyphenyl Acetic Acid
a-Hydroxyglutaric Acid-g-Lactone	Fumaric Acid	m-Inositol
a-Ketoglutaric Acid	g-Amino-N-Butyric Acid	Mono-Methylsuccinate
a-Methyl-D-Glucoside	g-Cyclodextrin	m-Tartaric Acid
a-Methyl-D-Mannoside	Gelatin	N-Acetyl-D-Galactosamine
Amygdalin	Gentiobiose	N-Acetyl-D-glucosaminitol
Arbutin	g-Hydroxybutyric Acid	N-Acetyl-L-Glutamic Acid
b-Cyclodextrin	Glucuronamide	Oxalic Acid
b-D-Allose	Gly-Asp	Oxalomalic Acid
b-Hydroxybutyric Acid	Glycine	Palatinose
b-Methyl-D-Galactoside	Glycogen	Pectin
b-Methyl-D-Glucoside	Glycolic Acid	p-Hydroxyphenyl Acetic Acid
b-Methyl-D-Glucuronic Acid	Glyoxylic Acid	Putrescine
b-Methyl-D-Xyloside	Hydroxy-L-Proline	Quinic Acid
b-Phenylethylamine	i-Erythritol	Salicin
Bromosuccinic Acid	Inulin	Sebacic Acid
Capric Acid	Itaconic Acid	Sec-Butylamine
Caproic Acid	Lactulose	Sedoheptulosan
Chondroitin Sulfate C	L-Alaninamide	Sodium butyrate
Citraconic Acid	Laminarin	Sorbic Acid
Citric Acid	L-Arabinose	Stachyose
D,L-Carnitine	L-Arabitol	Succinamic Acid
D,L-Citramalic Acid	L-Arginine	Succinic Acid
D,L-Malic Acid	L-Aspartic Acid	Sucrose
D,L-Octopamine	L-Fucose	Turanose
d-Amino Valeric Acid	L-Galactonic Acid-g-Lactone	Tween 20
D-Arabinose	L-Glucose	Tween 40
D-Arabitol	L-Glutamine	Tween 80
D-Aspartic Acid	L-Histidine	Tyramine
D-Cellobiose	L-Homoserine	Xylitol
